# Molecular epidemiology of *Blastocystis* isolated from animals in the state of Rio de Janeiro, Brazil

**DOI:** 10.1371/journal.pone.0210740

**Published:** 2019-01-25

**Authors:** Carolina Valença-Barbosa, Teresa Cristina Bergamo do Bomfim, Bernardo Rodrigues Teixeira, Rosana Gentile, Sócrates Fraga da Costa Neto, Bárbara Souza Neil Magalhães, Daniel de Almeida Balthazar, Fabio Alves da Silva, Renata Biot, Claudia Masini d’Avila Levy, Helena Lúcia Carneiro Santos

**Affiliations:** 1 Laboratório de Estudos Integrados em Protozoologia, Instituto Oswaldo Cruz/FIOCRUZ, Rio de Janeiro, RJ, Brazil; 2 Departamento de Parasitologia Animal da Universidade Rural do Rio de Janeiro, Seropédica, Rio de Janeiro, Brazil; 3 Laboratório de Biologia e Parasitologia de Mamíferos Silvestres Reservatórios, Instituto Oswaldo Cruz/FIOCRUZ, Rio de Janeiro, RJ, Brazil; 4 Fundação Jardim Zoológico da cidade do Rio de Janeiro, Parque da Quinta da Boa Vista, Rio de Janeiro, Brazil; 5 Instituto de Ciência e Tecnologia em Biomodelos/FIOCRUZ, Rio de Janeiro, Brazil; 6 Laboratório de Parasitologia do Departamento de Patologia do Hospital Universitário Antônio Pedro/Universidade Federal Fluminense, Niterói, Rio de Janeiro, Brazil; Faculty of Science, Ain Shams University (ASU), EGYPT

## Abstract

The enteric protist *Blastocystis* is one of the most frequently reported parasites infecting both humans and many other animal hosts worldwide. A remarkable genetic diversity has been observed in the species, with 17 different subtypes (STs) on a molecular phylogeny based on small subunit RNA genes (SSU rDNA). Nonetheless, information regarding its distribution, diversity and zoonotic potential remains still scarce, especially in groups other than primates. In Brazil, only a few surveys limited to human isolates have so far been conducted on *Blastocystis* STs. The aim of this study is to determine the occurrence of *Blastocystis* subtypes in non-human vertebrate and invertebrate animal groups in different areas of the state of Rio de Janeiro, Brazil. A total of 334 stool samples were collected from animals representing 28 different genera. *Blastocystis* cultivated samples were subtyped using nuclear small subunit ribosomal DNA (SSU rDNA) sequencing. Phylogenetic analyses and BLAST searches revealed six subtypes: ST5 (28.8%), ST2 (21.1%), ST1 and ST8 (19.2%), ST3 (7.7%) and ST4 (3.8%). Our findings indicate a considerable overlap between STs in humans and other animals. This highlights the importance of investigating a range of hosts for *Blastocystis* to understand the eco-epidemiological aspects of the parasite and its host specificity.

## Introduction

*Blastocystis* is a genus of unicellular anaerobic eukaryotic organisms in the diverse Stramenopiles group that includes brown algae, diatoms, slime nets and water molds [1–3]. It is a cosmopolitan enteric parasite and one of the most common protist parasites worldwide. The ability of *Blastocystis* to cause gastrointestinal and other diseases has been questioned, but the parasite undoubtedly possesses pathogenic potential although its virulence mechanisms are not well understood [4, 5].

*Blastocystis* is known to infect a wide range of animals including reptiles, invertebrates, birds, amphibians, humans and other mammals [6–11]. Recent studies on the small subunit ribosomal RNA (SSU-rDNA) gene identified at least 17 different *Blastocystis* subtypes (ST) in humans and a variety of animals including non-human primates (NHPs) and mammals, birds and insects [7, 8, 10–17]. All human *Blastocystis* isolates are currently classified into ten subtypes (ST1-ST9 and ST12), and all, except ST9, have also been identified in other animals [18, 19]. Subtypes ST10 to ST17 have been found exclusively in non-human hosts [7, 15, 20–22]. It has been proposed that *Blastocystis* can spread through human-to-human, animal-to-human and, possibly humans-to-animal contact [6, 15, 23–26]. It is still unclear whether animals may serve as reservoirs of *Blastocystis* strains colonizing humans. A higher risk of infection has been identified in animal handlers, supporting the hypothesis of transmission from animals to humans [6, 7, 9, 15, 27]. However, further evidence is still required in order to definitely confirm the occurrence of zoonotic transmission [7, 14, 15, 28].

While abundant data on the distribution and prevalence of *Blastocystis* STs in humans are available, there is scanty information about host specificity, genetic variation and distribution of STs in other hosts. Only a few surveys have been conducted in a limited number of animal groups from farms, national parks and zoos [11, 16, 29, 30]. In South America, data on *Blastocystis* STs remain limited especially in Brazil, where only a few studies have characterized *Blastocystis* isolates at the molecular level [31–37]. However, no surveys of the distribution and occurrence of *Blastocystis* in other non-human hosts have been conducted. Therefore, the aim of the present study was to determine the occurrence of *Blastocystis* STs among vertebrates and invertebrates in different areas of the state of Rio de Janeiro in southeastern Brazil.

## Materials and methods

### Samples

**Fig 1:**
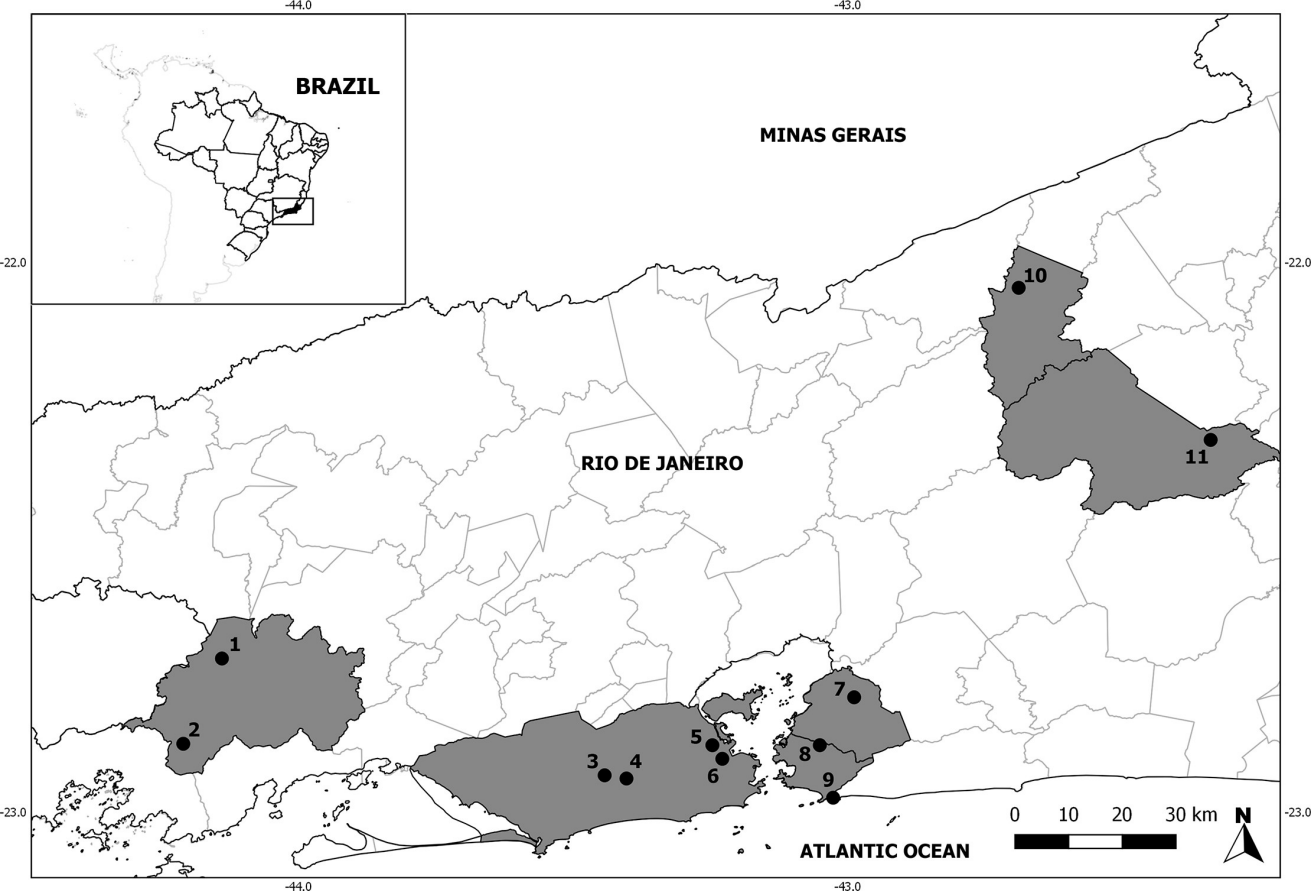
Geographical location of the 11 localities where stool samples from animals were collected.

Prior to data collection, the study protocol was reviewed and approved by the Committee of Ethics in Animal Experimentation of the Oswaldo Cruz Foundation Rio de Janeiro, Brazil (L–066/08; L–049/08, LW 81/12, LW-39/14) and permission for trapping and capture was granted by Brazilian Government’s Chico Mendes Institute for Biodiversity and Conservation (ICMBIO, license number 13373 and 46934-1) and the Environmental Institute of Rio de Janeiro State (INEA, license number 020/2011. Adult cockroaches were captured from drains in several urban dwellings. We collected fecal samples from captive breeding, domestic and wild animals in the state of Rio de Janeiro, Brazil. Samples from captive animals were collected at the Rio Zoo (ZOO) and the Institute of Biomodel Science and Technology (ICTB, Fiocruz), both in the municipality of Rio de Janeiro. Samples from domestic animals were collected in Santa Bárbara, Niterói municipality (NIT) (22°52'39.81"S 43° 3'4.16"W); Jardim Catarina, São Gonçalo municipality (SG) (22°47'26.41"S 42°59'19.23"W); and São Pedro da Serra, Nova Friburgo municipality (SPS) (22°19'21.43"S 42°20'23.02"W). Samples from wild animals were collected in: (i) Fiocruz Atlantic Forest Campus of the Oswaldo Cruz Foundation (CFMA) (22°56’18”S 43°24’11”W) and the Pedra Branca State Park (PEPB) (22°55’57”S 43°26’34”W), both in Rio de Janeiro municipality,(ii) Serra da Tiririca State Park (PEST), Niterói municipality (22°57’56.4”S 43°00’24.5”W), *(iii)* Encanto, Sumidouro municipality (SUMID) (22°02’46”S 42°41’21”W), and *(iv)* Lidice and Morro do Estado, Rio Claro municipality (22°52’30.6”S 44°12’34.9”W;22°43’12.5”S 44°8’21.6”W) (Fig 1).

We obtained fresh fecal samples from 89 non-human primates, two raccoons, 11 rodents, 26 marsupials, one armadillo, 57 birds, 39 swine, 96 cockroaches, and 13 reptiles. Feces of captive animals were collected from their cages to minimize contamination. In the case of rodents, marsupials and the armadillo, stools were collected in the field laboratory after dissection of viscera, stored in labeled plastic containers, and transported to the main laboratory.

### Culturing of fecal material

All samples were subjected to direct in vitro xenic cultivation of *Blastocystis* sp. using an aliquot of stool samples into 10 mL of Pavlova’s medium (1.29 g/L of Na_2_HPO_4_, 0.42 g/L of KH_2_PO_4_, 7.27 g/L of NaCl, 1.46 g/L of yeast extract) containing 10% heat-inactivated adult bovine serum (ABS), 10% of penicillin and streptomycin (1000 IU/mL and 500 μg/mL, respectively) and powdered rice starch. Xenic cultures were incubated at 37°C and examined every 2-7 days. Whenever the typical forms of the parasite were observed by standard light microscopic examination, suspensions were frozen at -20°C until DNA extraction. Cultures positive for *Blastocystis* were subcultured every three days and cells stored successfully in liquid nitrogen.

### DNA extraction and PCR

DNA extraction from cultured samples positive for *Blastocystis* was carried out using the Qiamp DNA Stool Mini Kit (Qiagen, Valencia, CA) according to manufacturer recommendations. DNA was stored at -20°C until use. The set of primers used in the PCR amplification of the SSU rDNA region were: forward Blast 505-532 (5’GGAGGTAGTGA CAATAAATC3’) and reverse Blast 998-1017 (5’TGCTTTCG CACTTGTTCATC3’) [8]. PCR reaction was performed in a final volume of 50 μL, contained 100 mM of Tris-HCl (pH 9.0), 500 mM of KCl, 1.5 mM of MgCl_2_, 200 μM each of dATP, dGTP, dCTP and dTTP; 0.2 μM of primer; 1.5 U of Platinum Taq DNA polymerase (Invitrogen Life Technologies, Carlsbad, CA, USA), 0.05% of bovine serum albumin (BSA) and 5 μL of DNA sample. The following reaction was used: denaturation and enzyme activation at 95°C for 5 min, 35 cycles of 95°C for 30 s, 55°C for 30 s, and 72°C for 2 s, followed by a final extension step of 72°C for 7 min. Obtained amplicons were loaded on 1.5% agarose gels in a Tris-borate Ethylenediaminetetraacetic acid buffer, run at 80 V for 60 min, stained with Gelred (Biotium Inc., Hayward, CA, USA) and then viewed under UV transillumination. PCR products were purified using the Wizard SV gel and PCR Clean-Up System kit (Promega, Madison, WI, USA), sequenced in both directions using the BigDye Terminator v.3.1 Cycle Sequencing Kit (Applied Biosystems), and run on the ABI 3730 Sequencing Platform.

Sequences were analyzed and edited using the SeqMan software (DNASTAR software package, DNASTAR Inc., Madison, WI, USA) and compared with previously published sequences using the BLASTN software from NCBI server (http://www.ncbi.nlm.nih.gov/BLAST). Other published sequences of different subtypes of *Blastocystis* from Genbank were downloaded, and multiple sequence alignment was performed using the Clustal W algorithm of the MEGA software version 6.0 [38]. Our consensus nucleotide sequences were deposited in the Genbank database under accession numbers MG280720-MG280771.

### Phylogenetic analysis

After alignment and trimming of 52 sequences, a 550-bp region was selected for the analyses. Sequence alignment was performed using two different probabilistic methods of phylogenetic analysis: maximum likelihood (ML) analysis performed in Phyml v.3.1 [39], and Bayesian inference analysis carried out using MrBayes v3.1.2 [40]. Statistical selection of the best-fit model of nucleotide substitution in the SSU-rDNA gene was performed using the program jModelTest version 2.1.7 [41] and based on the Akaike Information Corrected Criterion (AICC) and Bayesian Information Criterion (BIC) for ML and BI analysis respectively.

For both analyses, the model of nucleotide substitution defined as the best fitted to the data was HKY [42] + G (gamma distribution of rates with four rate categories) with four free parameters and unequal base frequencies (AICc= 6626.915359; -lnL=2948.68156 and BIC= 7018.998707; -lnL=2948.68156). The phylogenetic trees were constructed and rooted using *Proteromonas lacertae* as the outgroup, due to its close relationship with *Blastocystis* [1, 14]. For ML, bootstrap samplings with 1.000 replicates were carried out to assess the branch reliability. The BI analysis was done for 10 million generations with sampling trees every 100th generation. Bayesian posterior probabilities were calculated using a Markov chain Monte Carlo sampling approach with four chains. The first 25% of the sampled trees were discarded as burn-in for each data set and convergence was assessed by evaluating the average standard deviation of split frequencies, which were below the recommended values (<0.01).

## Results

We analyzed 334 stool samples from 28 different animal genera. Overall occurrence of *Blastocystis* in fecal cultures was 34.4% (115/334) or 1/1 (100%) in the case of the armadillo, 21/26 (81%) in marsupials, 30/39 (77%) in pigs, 9/13 (69%) in reptiles, 7/11(64%) in rodents, 33/89 (37%) in NHPs, 12/57 (21%) in birds, and 2/96 (2%) in cockroaches (Table 1).

**Table 1:** *Blastocystis* positive samples and subtypes identified from vertebrates and invertebrates.

Host group	Location	Scientific name	N°	Pos	*Blastocystis* subtype (ST)					
					ST1	ST2	ST3	ST4	ST5	ST8
**Nonhuman primates**	ZOO	*Cebus apella xanthosternos*	3	0	-	-	-	-	-	-
		*Macaca nemestrina*	1	0	-	-	-	-	-	-
		*Ateles* sp	1	1	-	-	-	-	-	1
		*Chlorocebus* sp	2	0	-	-	-	-		-
		*Leontopithecus chrysomelas*	2	0	-	-	-	-	-	-
		*Lagothrix lagotricha *	2	1	-	-	-	-	-	1
		*Saguinus imperator*	6	0	-	-	-	-	-	-
		*Papio* sp	3	1	-	-	1	-	-	-
		*Pan troglodytes*	1	1	-	1	-	-	-	-
		*Alouatta* sp	2	1	-	-	-	-	-	1
		*Macaca fuscata*	2	1	1	-	-	-	-	-
		*Aotus *sp	4	2	1	-	-	-	-	1
	ICTB	*Macaca mulatta *	30	18	-	9	1	-	-	2
		*Saimiri sciureus*	10	0	-	-	-	-	-	-
		*Macaca fascicularis*	20	7	-	1	1	-	-	-
**Raccoon**	ZOO	*Procyon lotor*	2	0	-	-	-	-	-	-
**Rodents**	CFMA	*Akodon cursor*	1	1	-	-	-	-	-	-
		*Rattus rattus*	1	1	-	-	1	-	-	-
	Rio Claro	*Akodon cursor*	1	0	-	-	-	-	-	-
		*Akodon montensis*	2	2	-	-	-	-	-	-
		*Oligoryzomys nigripes*	1	0	-	-	-	-	-	-
		*Nectomys squamipes*	1	1	-	-	-	-	-	-
	SUMID	*Nectomys squamipes*	2	2	-	-	-	-	-	1
	PEST	*Oligoryzomys nigripes*	1	0	-	-	-	-	-	-
		*Trinomys eliasi*	1	0	-	-	-	-	-	-
**Marsupials**	CFMA	*Metachirus nudicautatus*	1	1	1	-	-	-	-	-
		*Didelphis aurita*	10	9	3	-	-	-	-	1
	PEPB		7	3	-	-	-	-	-	-
	Rio Claro		8	8	-	-	-	-	-	-
**Armadillo**	SUMID	*Dasypus septemcinctus*	1	1	-	-	-	-	-	1
**Birds**	NIT	*Gallus gallus*	12	1	-	-	-	-	1	-
		*Anas domesticus*	4	1	-	-	-	-	-	-
	ZOO	*Anas domesticus / Cairina moschata*	40	9	-	-	-	-	-	-
	SPS	*Anser anser*	1	1	1	-	-	-	-	-
**Swine**	SG	*Sus scrofa*	27	22	-	-	-	-	12	1
	SPS		12	8	3	-	-	1	2	-
**Cockroaches**	NIT	*Periplaneta americana*	60	2	-	-	-	1	-	-
	RJ		36	0	-	-	-	-	-	-
**Reptiles**	ZOO	*Chelonoidis *sp	13	9	-	-	-	-	-	-
**Total**			334	115	10	11	4	2	15	10

N°= number; Pos=Positive

Out of the 115 cultures, 70.4% (81/115) were confirmed by PCR. Of these samples, 52 were successfully subtyped by sequence analyses at the SSU-rRNA gene. A total of 29 samples were untypable due to poor sequence quality (N= 20) and sequencing overlapping (N=9). BLAST search and phylogenetic analysis identified six subtypes. The most common subtype was ST5 (28.8%), followed by ST2 (21.1%), ST1 (19.3%), ST8 (19.3%), ST3 (7.7%) and ST4 (3.8%). All but three DNA sequences showed high identity (99%-100%) with their closest matching reference sequences of *Blastocystis* from Genbank, thus allowing direct subtyping of the corresponding isolates. The DNA sequences ZOO6 and ICTB_AG149 presented 98% identity, while ZOO7 showed 97% identity with reference sequences.

Regarding the distribution of *Blastocystis* STs in this study, ST1, ST2, ST3, and ST8 were identified in NHPs. In swine, isolates were typed as ST1, ST4, ST5, and ST8. We found ST3 and ST8 in two species of rodents, ST1 and ST8 in opossums, and ST8 in the armadillo. Birds were infected with *Blastocystis* ST1 and ST5 while the cockroach was infected with ST4 (Table 1).

**Fig 2:**
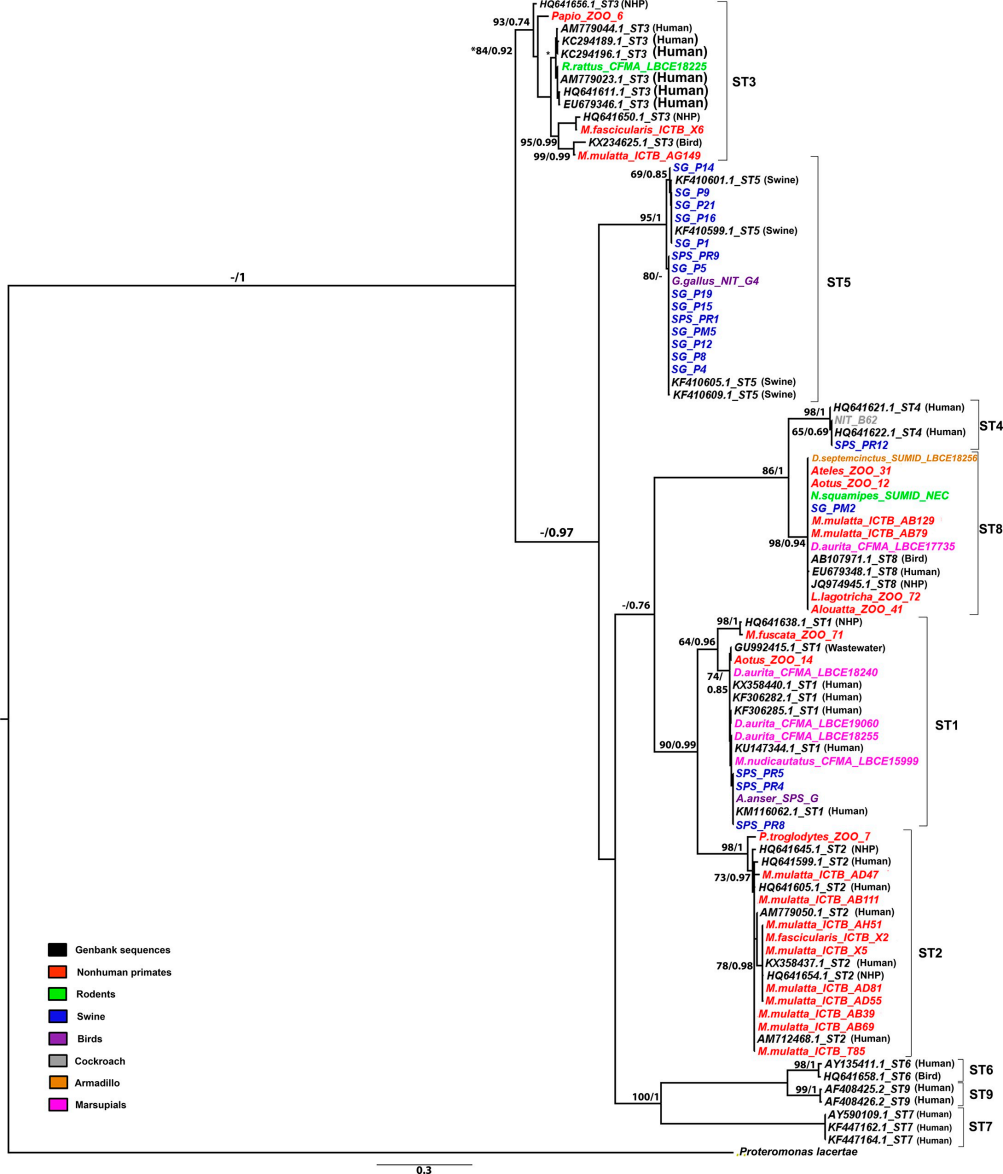
Maximum likelihood (ML) and Bayesian inference reconstruction tree of *Blastocystis* based on SSU-rDNA gene using sequences obtained in the study and Genbank retrieved. The first number associated with each node represents the ML bootstrap value (values below 50% were not shown) followed by the Bayesian posterior probabilities. The scale bars indicate the expected number of substitutions per site.

Both rooted trees (ML and BI) showed eight clades that corresponded exactly to ST1 to ST8. Each subtype clade was strongly supported by high bootstrap values and Bayesian posterior probabilities. Moreover, some isolates formed small clades within each subtype, showing intra-strain variation (Fig 2). The two phylogenetic trees were topologically identical.

## Discussion

This is the first study of *Blastocystis* subtypes in a variety of non-human animal hosts in Brazil. *Blastocystis* has a wide host range, with subtypes overlapping in several animal genera. Our study highlights the large genetic diversity between *Blastocystis* isolates from animals, provides the first molecular evidence from armadillo and *Nectomys squamipes*, and of the presence of different *Blastocystis* subtypes in cockroaches (ST4), rodents (ST8), pigs (ST4 and ST8).

In such species, we found *Blastocystis* STs that had been previously identified in humans. We isolated ST1 and ST5 in samples respectively from a goose and a chicken collected in a farm. This can be explained by the fact that the caregivers left the birds free during the day, allowing contact with other farm and domestic animals and favoring the circulation of *Blastocystis* subtypes. It is also important to note that these animals also maintain close contact with humans. ST6 and ST7 have been conceived as typical “avian subtypes” [14, 43, 44]; however, none of these STs was observed in our study. Considering invertebrates, we obtained only two *Blastocystis*-positive samples from cockroaches. One isolate was identified as ST4, which we have identified for the first time in this species [10, 45]. Previous cockroaches isolates were identified as ST3 [46].

In general, ST4 is the most frequent ST in rodents, despite not being found in all rodent species [11, 29]. ST1, ST3, ST5, ST10, and ST17 were also found in rodents in previous investigations [7, 11, 30]. We detected ST3 in *Rattus rattus*, and also ST8 for the first time in the South American water rat *Nectomys squamipes*, thus expanding the number of subtypes recorded in rodents. ST8 had already been found in humans in Sumidouro, Brazil [36], confirming its circulation in both human and animal populations in this region. *N*. *squamipes* inhabits the banks of streams, rivers and flooded areas, and has a wide geographical distribution in Brazil [47]. It has been reported as an important source of intestinal parasites infections such as *Schistosoma mansoni* in Sumidouro [48, 49]. According to official reports (SEA, 2014) [50], almost 80% of the population in this region has no access to appropriate sewage services and rely on effluent disposal (rudimentary trench, ditch, river, lake, and other types), which may lead to fecal contamination of soil and water.

We also identified *Blastocystis* ST8 for the first time in an armadillo. This species is widely distributed in South America [51–53]. In the sampled marsupials, we observed elevated levels of *Blastocystis* infection by ST1 and ST8 in two species (*Didelphis aurita* and *Metachirus nudicaudatus*). The black-eared opossum *D*. *aurita* is a generalist and opportunist marsupial, very abundant in degraded areas of the Atlantic Forest [54, 55], possibly explaining the high prevalence of *Blastocystis*. This is also the first report of *Blastocystis* infection in another marsupial (*Metachirus nudicaudatus*). This species is primarily insectivorous but also feeds on small vertebrates, seeds and fruits [55, 56, 57], increasing the chances of ingesting *Blastocystis* cysts. Further studies of native Brazilian fauna should increase our knowledge of *Blastocystis* epidemiology and host specificity.

*Blastocystis* infection is common in domestic pigs [58, 59], primarily by the two subtypes ST1 and ST5 [7, 10, 12, 21, 60, 61]. However, six *Blastocystis* STs have been reported in pigs worldwide (ST1-ST3, ST5-ST7), with ST5 as the most common [8, 10, 12, 14, 15, 21, 25, 29, 62]. ST5 was the most prevalent in our samples. We have expanded the number of subtypes recorded in pigs by identifying ST4 and ST8. ST4 was isolated from a pig sampled from a farm, while ST8 was identified in a slum. Pigs bred under unhealthy conditions can be an important source of transmission of several parasites including *Blastocystis*. Previous studies demonstrated *Blastocystis* transmission from animals under human care, including pigs [6, 7, 15, 28, 60, 63], which indicates the potential for zoonotic transmission by pigs, as previously suggested by the detection of the zoonotic subtype ST5 in both pigs and piggery workers in Queensland, Australia [15, 25].

We identified ST1-ST3 and ST8 in non-human primates, with ST2 as the predominant subtype, in agreement with previous screens [6, 7, 15, 16, 29, 64, 65]. ST8 is common in NHP caregivers, further supporting a possible zoonotic spread of *Blastocystis* from to humans [7]. NHPs are often infected by *Blastocystis* with ST1, ST2 and ST3 being the most frequent subtypes both in NHPs and humans across the world [9, 16, 29, 34, 65–68]. Considering that the most surveys were conducted with captive NHPs [6, 7, 11, 15, 64, 65] in close contact with humans and that ST1-3 can circulate in both hosts, it seems reasonable to assume the possibility of zoonotic transmission. *Blastocystis* transmission between animals can also occur through interactions in zoos, resulting in the potential transmission of distinct STs among animals and to humans [6, 15] Nonetheless, *Blastocystis* subtype data obtained from zoos and labs should be interpreted with care since captive and wild populations may be exposed to different STs [29].

*Blastocystis* has been previously cultured in a wide range of media. In general, any medium developed for the isolation and growth of *Entamoeba spp*. will also support *Blastocystis*. The widely used Jones’ medium is ideal for short term culturing of multiple subtypes [69, 70]. However, xenic cultures of *Blastocystis* seem to be relatively stable irrespective of source and medium reagents. For this reason, in our study Pavlova medium was used to successfully identify *Blastocystis* in various animal species.

Despite our considerable effort in analyzing 115 culture samples, one limitation of our study was that no amplification was observed in 34 samples. We choose to work with cultured samples to avoid DNA extraction from lower parasite densities or problems with PCR inhibitors in feces. PCR generally works better with DNA extracted from *Blastocystis* cultures than directly from feces. Our primers targeted a conserved region of the SSU rDNA gene but were designed by Santin et al. [8] based on a relatively small number of animals (NHPs, swine, cattle, and chicken). One intriguing possibility suggested by our data is that some infections may go undetected when those primers are used. However, it is also clear that some subtypes exhibit substantial genetic diversity, and thus the ability of primers to detect all genetic variants is yet to be determined. Applying other primers to the 34 *Blastocystis* samples that failed to be amplified may provide an answer to this question, and to this purpose, we have planned a future comparative study of the techniques.

Moreover, there are intrinsic obstacles to accurately assessing mixed infections through the employed methodologies. Subtyping of isolates in mixed infections requires cloning of the PCR product and sequencing of several clones. Investigations on mixed infections are therefore rare. Only one study that has investigated *Blastocystis* mixed infections in humans [71]. The study analyzed 50 clones and detected three different subtypes of *Blastocystis* in humans (ST2, ST3, and ST4). However, their analyses were based on a single individual, thus restricting interpretations. Preparation of multiple clone libraries is laborious and prohibitive, and it is not clear how many clones are needed to identify mixed infections. In addition, levels of diversity of *Blastocystis* within a host have never been investigated.

Lastly, in agreement with previous findings [29, 72], the high level of genetic variability found in ST1, ST2, and ST3 seems to support the low host specificity of these subtypes. Although ST1 and ST3 are predominant in humans, both have been found in a wide range of animals. Our findings show that not only ST1, ST2 and ST3, but also ST4, ST5, and ST8 were shared by many hosts, including non-mammals, suggesting the circulation of these subtypes in different animal populations. These results support the low host-specificity of *Blastocystis* and cross-infectivity among distinct hosts. Most isolates from animals and humans are genetically similar or identical, providing further evidence for cross-transmission [6, 15].

## Conclusions

Our results indicate a considerable overlap between *Blastocystis* subtypes across in different hosts. Future studies should extend our findings need and investigate a larger number of samples and animal orders, so as to better understand the ecology, epidemiology and host specificity of *Blastocystis*. Our findings also further contribute to defining the genetic characteristics of *Blastocystis* in different hosts in Brazil and other countries.

## Supporting information

S1 Table *Blastocystis* isolates obtained in the present study. (DOCX)
