# Supplementary material for: Molecular epidemiology of Blastocystis isolated from animals in the state of Rio de Janeiro, Brazil
Source: PLoS One. 2019 Jan 25;14(1):e0210740. doi: 10.1371/journal.pone.0210740 (PMC6347289; doi:10.1371/journal.pone.0210740)
Supplement: S1 Table — (DOCX) [file pone.0210740.s001.docx]

| **Subtype** | **Identity**  **(%)** | **N° of isolates** | **Isolate code and Genbank accession number** |
| --- | --- | --- | --- |
| **ST1** | **99-100%** | **10** | SPS_G (MG280720), SPS_PR4 (MG280721), SPS_PR5 (MG280722), SPS_PR8 (MG280723), LBCE_15999 (MG280724), LBCE_18255 (MG280725), LBCE_18240 (MG280726), LBCE_19060 (MG280727), Zoo_71 (MG280728), Zoo_14 (MG280729) |
| **ST2** | **97-100%** | **11** | Zoo_7 (MG280730), ICTB_X5 (MG280731), ICTB_X2 (MG280732), ICTB_T85 (MG280733), ICTB_AH51 (MG280734), ICTB_AD81 (MG280735), ICTB_AD55 (MG280736), ICTB_AD47 (MG280737), ICTB_AB69 (MG280738), ICTB_AB39 (MG280739), ICTB_AB111 (MG280740) |
| **ST3** | **98-99%** | **4** | ICTB_X6 (MG280741), LBCE_18225 (MG280742), Zoo_6 (MG280743), ICTB_AG149 (MG280744) |
| **ST4** | **100%** | **2** | SPS_PR12 (MG280745), NIT_B62 (MG280746) |
| **ST5** | **99-100%** | **15** | SG_P1 (MG280747), SG_P4 (MG280748), SG_P8 (MG280749), SG_P12 (MG280750), SG_PM5 (MG280751), SPS_PR1 (MG280752), SG_P14 (MG280753), SG_P15 (MG280754), SG_P16 (MG280755), SG_P19 (MG280756), SG_P21 (MG280757), NIT_G4 (MG280758), SG_P9 (MG280759), SG_P5 (MG280760), SPS_PR9 (MG280761) |
| **ST8** | **100%** | **10** | LBCE_17735 (MG280762), ITCB_AB79 (MG280763), ICTB_AB129 (MG280764), SG_PM2 (MG280765), NEC (MG280766), Zoo_12 (MG280767), Zoo_31 (MG280768), LBCE_18256 (MG280769), Zoo_72 (MG280770), Zoo_41 (MG280771) |

**Table 1:** *Blastocystis* isolates obtained in the present study
